# Decreasing central-line blood draws by consolidation of phlebotomy timing: results of a quality improvement project

**DOI:** 10.1186/cc13277

**Published:** 2014-03-17

**Authors:** G Arteaga, S Tripathi, Y Ouellette, M Nemergut, G Rohlik, K Fryer, C Huskins

**Affiliations:** 1 Mayo Clinic, Rochester, MN, USA

## Introduction

The direct central line entry rate is believed to be a major contributor to the risk of central line infections. At Mayo Clinic there was historically no schedule for obtaining blood for analysis in the pediatric ICU. A policy was implemented in May 2013 to restrict blood draws to three times daily for nonemergent blood draws only. We subsequently conducted this study to determine whether implementation of this policy was associated with a reduction of blood draws as well as central-line unique entries.

## Methods

Data from the laboratory as well as database for Central Line Unique Entry were analyzed at baseline and after implementation of the policy change for identification of any decrease in line entry/blood draw rate. As per Mayo Clinic policy, IRB approval was not required for a QI project.

## Results

In the pre-implementation phase there were a total of 4,602 blood draws in 5,227 total patient-days, (0.88 blood draws/patient- days). After consolidation, there were 1,095 blood draws in 1,491 patient-days (0.73 blood draws/patient-day; 17% reduction). Of these line entries, 24.7% were arterial line entry, 50.5% central line entry and 12.5% were by peripheral venipuncture. After policy implementation, these numbers were 10.9%, 49.7%, and 23.8%, respectively. The average central line unique entry after blood draw consolidation decreased from 10 to 6 line entries/central line-day. Consolidation of blood draws was associated with a cost saving of $7,200/year.

## Conclusion

Consolidating time frames for blood draws in the PICU was associated with decreased central line entries, decreased utilization of vascular access teams, and decreased phlebotomy cost. We hypothesize that this policy will be associated with a decreased incidence of CLABSI when more patients are included for analysis.

